# Delayed-onset heparin-induced thrombocytopenia presenting with multiple arteriovenous thromboses: case report

**DOI:** 10.1186/1752-1947-1-131

**Published:** 2007-11-10

**Authors:** Abbas Salehi Omran, Abbasali Karimi, Hossein Ahmadi, Parin Yazdanifard

**Affiliations:** 1 Cardiovascular Surgery Department, Tehran Heart Center, Medical Sciences/University of Tehran, Iran; 2 Clinical Reaserch Department, Tehran Heart Center, Medical Sciences/University of Tehran, Iran

## Abstract

**Background:**

Delayed-onset heparin-induced thrombocytopenia with thrombosis, albeit rare, is a severe side effect of heparin exposure. It can occur within one month after coronary artery bypass grafting (CABG) with manifestation of different thrombotic events.

**Case presentation:**

A 59-year-old man presented with weakness, malaise, bilateral lower limb pitting edema and a suspected diagnosis of deep vein thrombosis 18 days after CABG. Heparin infusion was administered as an anticoagulant. Clinical and paraclinical work-up revealed multiple thrombotic events (stroke, renal failure, deep vein thrombosis, large clots in heart chambers) and 48 ×10^3^/μl platelet count, whereupon heparin-induced thrombocytopenia was suspected. Heparin was discontinued immediately and an alternative anticoagulant agent was administered, as a result of which platelet count recovered. Heparin-induced thrombocytopenia, which causes thrombosis, is a serious side effect of heparin therapy. It is worthy of note that no case of delayed-onset heparin-induced thrombocytopenia with thrombosis associated with cardiopulmonary bypass surgery has thus far been reported in Iran.

**Conclusion:**

Delayed-onset heparin-induced thrombocytopenia should be suspected in any patient presenting with arterial or venous thromboembolic disorders after recent heparin therapy, even though the heparin exposure dates back to more than a week prior to presentation; and it should be ruled-out before the initiation of heparin therapy.

## Background

Heparin-induced thrombocytopenia (HIT) is a severe side effect of heparin and is associated with heparin-platelet factor (PF4) antibodies. The incidence of HIT in patients who have undergone cardiopulmonary bypass surgery has been estimated at 1.9% [1]. HIT causes not only thrombocytopenia but also arterial and venous thrombotic events such as lower limb ischemia, stroke, acute myocardial infarction and renal failure [2,3]. The time course to development of HIT is generally within 4 to 14 days after the initiation of heparin therapy.

Recently, however, it has been shown that HIT can develop in a delayed fashion, i.e. 1 to 3 weeks after the cessation of heparin [4].

We present a descriptive case of a patient who developed heparin-induced thrombotic thrombocytopenia (HITT) 18 days after CABG, resulting in multiple arteriovenous thromboses.

## Case history

Our patient, a 59-year-old man with past history of diabetes mellitus and hypertension, presented with signs and symptoms of acute coronary syndrome for several hours prior to Cardiac Care Unit (CCU) admission. At CCU, the patient was diagnosed with acute coronary syndrome, and drugs including unfractionated heparin (UFH) were administered. An angiography of his coronary vessels showed three-vessel disease and a left ventricular ejection fraction (LVEF) of about 35%.

He underwent CABG urgently with four grafts on his diseased coronary vessels, including left internal mammary artery graft and 3 saphenous vein grafts. We did not use any glycoprotein IIb-IIIa receptor antagonist during operation. The patient was weaned from cardio pulmonary bypass uneventfully.

During the patient's hospital stay, there were no clinical features suggestive of HITT or any other abnormalities in his platelet count and other laboratory tests. He was discharged to his home on the eleventh postoperative day in good condition with a platelet count of 139 × 10 ^3^/μl, LVEF of about 35% and normal levels of creatinine and urea.

**Figure 1:**
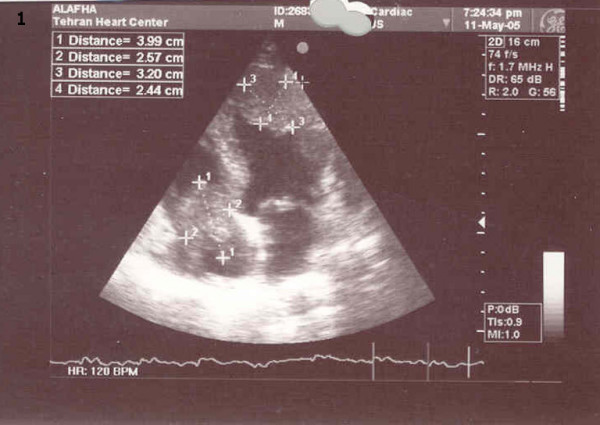
Right atrium (3.99 × 2.57 mm), Right ventricle and left ventricle Clots (3.20 × 2.44 mm).

**Figure 2:**
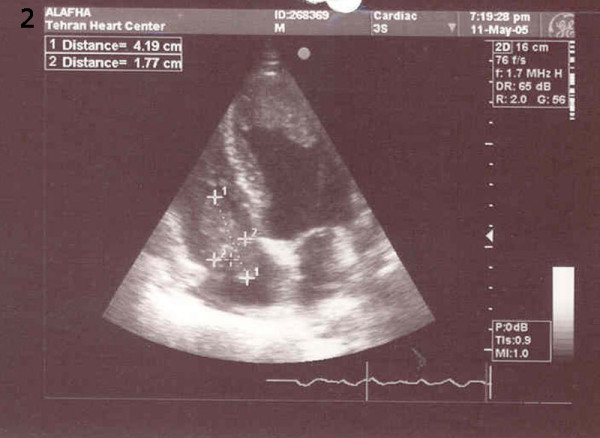
Right atrium (3.99 × 2.57 mm), Right ventricle and left ventricle Clots (3.20 × 2.44 mm).

One week later (18 days after surgery), the patient returned to our hospital complaining of weakness, malaise and bilateral lower limb pitting edema for the previous two days; he was, subsequently, readmitted to surgical ward. UFH heparin infusion was commenced with the diagnosis of deep vein thrombosis (DVT). Work-up showed a low platelet count of about 48 × 10 ^3^/μl, prolonged active partial thromboplastin time of 75 seconds, DVT of the left lower limb, ejection fraction of about 25–30% with very large mobile clots in the right atrium (40 × 20 mm), as well as in the left ventricle and pulmonary artery (Fig 1, 2). Brain Computerized Tomography scans showed cortical infarction and deep white matter ischemia in the right temporoparietal lobe. The patient's creatinine and urea levels were about 3.6 mg/dl and 114 mg/dl, respectively. HITT being suspected as the culprit, heparin infusion was interrupted; and because of the unavailability of lepirudin or other alternative anticoagulants, warfarin was started. Serial lab data showed a gradual rise in platelet count, and two weeks later his platelet count and aPTT were within normal ranges.

**Figure 3:**
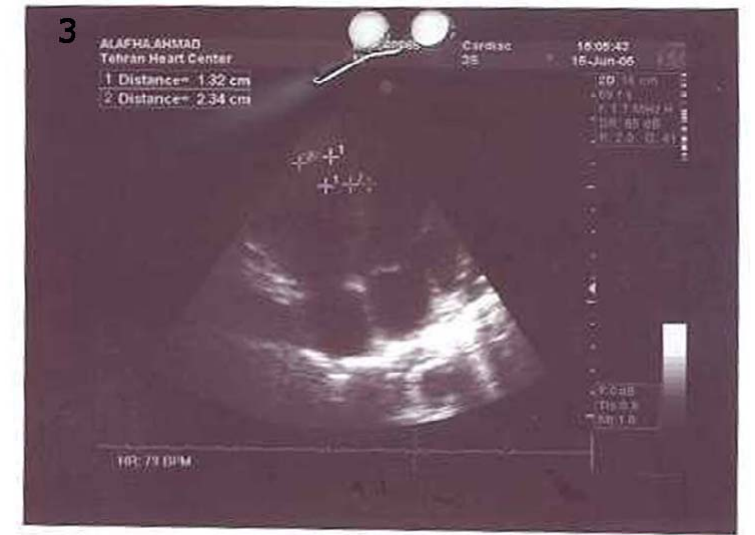
echocardiography shows disappearing of clots after treatment.

**Figure 4:**
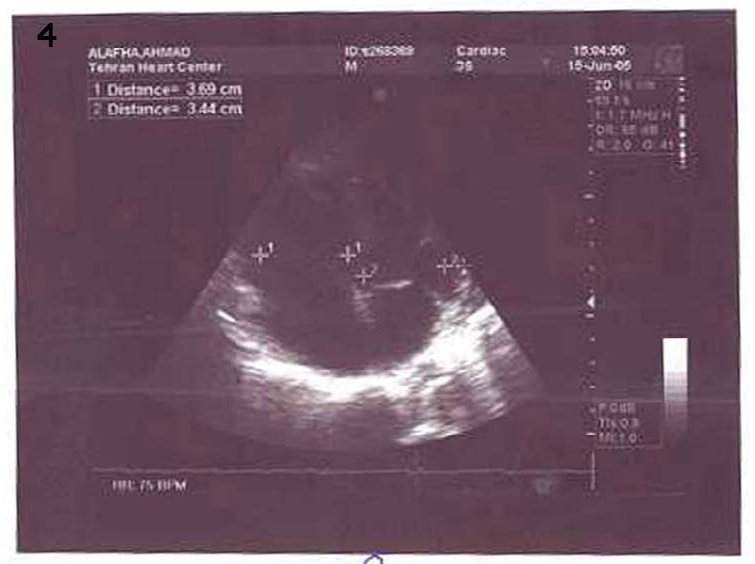
echocardiography shows disappearing of clots after treatment.

LVEF was 35% with mild tricuspid regurgitation without any clotting in the left ventricle, right atrium or pulmonary artery. Pitting edema had decreased, but on account of the fact that the patient's creatinine level was 3.2 mg/dl, hemodialysis without heparin was performed so as to decrease the creatinine level. The patient was discharged one month after re-admission in good condition with no clinical signs and symptoms and with disappeared clots (Fig 3 and 4). Further follow-up of the patient at one year did not reveal any clinical or paraclinical features suggestive of DVT due to warfarin therapy for HITT [5].

## Discussion

HIT (heparin-induced thrombocytopenia) and HITT (heparin-induced thrombotic thrombocytopenia) are different manifestations of the same immune disease; during these conditions, the body releases IgG antibody (immune globulin G) against heparin-PF4 complex.

The incidence of HIT in patients who have undergone cardiopulmonary bypass surgery has been estimated at 1.9% [1].

HITT presents with signs and symptoms of the involvement of multi ranges due to vascular thrombosis in those areas, such as cerebral vascular accidents, myocardial infarction and limb ischemia.

When HITT develops postoperatively, heparin must be discontinued and alternative anticoagulant agents given. Alternative anti coagulant agents are intravenous hirudin, argatroban, danaproid or lepirudin [6-8]. Warfarin is recommended to start simultaneously with hirudin or other alternative anticoagulants except heparin. Warfarin is not contraindicated in treatment of HITT but because of its potential to make limb gangrene besides of heparin, it is recommended to administer after other anticoagulant in HITT [5,8].

In our case, the unavailability of alternate anticoagulants like hirudin obliged us to start only oral warfarin and conservative management without platelet transfusion, to which our patient responded very well.

Diagnosis of HITT is clinical although it must be confirmed by serological tests. In our case despite of unavailability of confirmatory tests such as heparin-platelet factor (PF4) antibodies, typical manifestation of multiorgan involvement leads us to make diagnosis of HITT.

The time course to development of HIT is generally within 4 to 14 days after the initiation of heparin therapy. Nonetheless, it has recently been demonstrated that HIT can develop in a delayed fashion, i.e. 1 to 3 weeks after the cessation of heparin [4].

Our experience highlights the advisability of the evaluation of any thromboembolic event occurring within 1 month of hospital discharge after CABG for possible delayed-onset HITT before treatment with heparin.

Overall, it is expedient that the impression of HIT and HITT after CABG be treated with due sensitivity and that in cardiac surgical patients the platelet count be carefully monitored after the operation. A fall of 50% or greater from the highest postoperative count (with or without thrombosis) is suggestive of HIT [9].

## Conclusion

Delayed-onset HIT should be suspected in any patient presenting with arterial or venous thromboembolic thromboembolic disorders after recent heparin therapy, even though the heparin exposure is more than a week before representation. Delayed-onset HIT should be considered and ruled-out before initiating heparin therapy in this group of patients.

## List of abbreviations

**CABG **(coronary artery bypass grafting)

**CCU **(Cardiac Care Unit)

**DVT **(deep vein thrombosis)

**HIT **(heparin-induced thrombocytopenia)

**HITT **(heparin-induced thrombotic thrombocytopenia)

**LVEF **(left ventricular ejection fraction)

**PF4 **(heparin-platelet factor)

## Competing interests

We have not received reimbursements, fees, funding or salary from any organization and do not hold any stocks or shares in any organization that may in any way gain or lose financially from the publication of this manuscript, neither now nor in the future.

We are not currently applying for patents and have not received any reimbursements, fees, funding, or salary from any organization that holds or has applied for patents relating to the content of the manuscript.

We have not any financial or non-financial competing interests (political, personal, religious, ideological, academic, intellectual, commercial or any other) relating to the content of this manuscript.

## Authors' contributions

**A S **carried out the surgery, managed postoperative complication, and was directly involved in conception, design and drafting the manuscript. **A K **and **H A **gave critical comments on the results and participated in planning and coordination of the study. **P Y **participated in the design of study, and was directly involved in drafting and revising the manuscript. All Authors read and approved the final manuscript.

## Consent

Written consent was obtained from the patient for publication of this study.
